# A Next-Generation Sequencing Approach Uncovers Viral Transcripts Incorporated in Poxvirus Virions

**DOI:** 10.3390/v9100296

**Published:** 2017-10-13

**Authors:** Marica Grossegesse, Joerg Doellinger, Berit Haldemann, Lars Schaade, Andreas Nitsche

**Affiliations:** 1 Centre for Biological Threats and Special Pathogens, Highly Pathogenic Viruses (ZBS 1), Robert Koch Institute, Seestr. 10, 13353 Berlin, Germany; GrossegesseM@rki.de (M.G.); DoellingerJ@rki.de (J.D.); 2 Centre for Biological Threats and Special Pathogens, Proteomics and Spectroscopy (ZBS 6), Robert Koch Institute, Seestr. 10, 13353 Berlin, Germany; 3 Methodology and Research Infrastructure, Bioinformatics (MF 1), Robert Koch Institute, Nordufer 20, 13353 Berlin, Germany; HaldemannB@rki.de; 4 Centre for Biological Threats and Special Pathogens (ZBS), Robert Koch Institute, Nordufer 20, 13353 Berlin, Germany; SchaadeL@rki.de

**Keywords:** transcript incorporation, poxvirus, cowpox virus, next-generation sequencing

## Abstract

Transcripts are known to be incorporated in particles of DNA viruses belonging to the families of *Herpesviridae* and *Mimiviridae*, but the presence of transcripts in other DNA viruses, such as poxviruses, has not been analyzed yet. Therefore, we first established a next-generation-sequencing (NGS)-based protocol, enabling the unbiased identification of transcripts in virus particles. Subsequently, we applied our protocol to analyze RNA in an emerging zoonotic member of the *Poxviridae* family, namely Cowpox virus. Our results revealed the incorporation of 19 viral transcripts, while host identifications were restricted to ribosomal and mitochondrial RNA. Most viral transcripts had an unknown and immunomodulatory function, suggesting that transcript incorporation may be beneficial for poxvirus immune evasion. Notably, the most abundant transcript originated from the *D5L/I1R* gene that encodes a viral inhibitor of the host cytoplasmic DNA sensing machinery.

## 1. Introduction

Transcripts have been shown to be incorporated in virions of dsDNA viruses such as cytomegalovirus (CMV) [1,2,3], herpes simplex virus 1 (HSV-1) [4] and mimivirus [5]. While only viral transcripts have been detected in mimivirus particles [5,6], CMV and HSV-1 particles contained also host transcripts [1,2,3,4]. Intact virion-packaged transcripts are released upon entry into the host cell, enabling the rapid expression of according proteins [2,4]. However, the incorporation of mRNA in virus particles appears to occur in proportion to the intracellular concentration during virus assembly [1,3]. Moreover, it has been shown for CMV that transcript incorporation is independent of sequence-specific *cis*-acting packaging elements as present, e.g., in retroviruses [3]. Instead, transcript packaging into virions may rather be mediated by nonspecific interactions of mRNA with virion proteins [7]. The biological function of virion-incorporated transcripts has barely been investigated so far. Nevertheless, interestingly, mRNA encoding a secreted viral chemokine receptor, which binds and inhibits RANTES (regulated on activation, normal T cell expressed and secreted) chemokine, was identified in CMV virions [3,8], suggesting immunomodulatory advantages mediated by virion-incorporated transcripts. Although transcript incorporation is presumably an undirected process, virion-packaged mRNA may be regulated by mRNA levels during virus assembly, as suggested by Terhune and colleagues [3]. In this way, transcript incorporation may also contribute to virus adaption.

Except for viruses belonging to the families of *Herpesviridae* [1,2,3,4] and *Mimiviridae* [5], no other DNA virus particles have been analyzed yet for mRNA incorporation. The family of *Poxviridae* comprises highly complex dsDNA viruses that encode more than 200 genes. Poxvirus replication, transcription and assembly occur in distinct cytoplasmic areas called virus factories [9]. Because of the close proximity of these processes, it seems to be an attractive hypothesis that poxvirus particles package transcripts. In the present study, cowpox virus (CPXV), which belongs to the genus orthopoxvirus (OPV), was used as a model poxvirus to analyze transcript incorporation. Although wild rodents are the natural reservoir of CPXV [10], these viruses are capable of infecting a wide range of host species, including humans [11]. The number of zoonotic CPXV infections in Europe is increasing [12], but mechanisms of pathogenicity and host range are not yet completely understood. CPXV encode several mRNA-interacting proteins, including proteins for polyadenylation, capping and decapping [13], which have also been identified in highly pure CPXV mature virions [14]. Hence, packaging of these proteins into virions may result in co-packaging of mRNA.

Virion-incorporated RNAs have been analyzed in previous studies using gene array technology, PCR and nucleic-acid-sequence-based amplification (NASBA) [1,2,3,4,5]. In order to identify RNA in poxvirus particles, we first established a next-generation-sequencing (NGS)-based protocol, which has several advantages over the previously used methods. The “open view” of NGS enables the identification of both viral and host transcripts and, moreover, readily enables the identification of intact transcripts and possible genomic DNA contaminations. Additionally, relative quantitative information about incorporated transcripts is obtained. We propagated CPXV in HEp-2 and Rat-2 cell lines and purified mature virions by established sucrose and caesium chloride (CsCl) gradient ultracentrifugation. RNA in virions was analyzed by different sample preparation protocols prior to NGS, revealing primarily the incorporation of viral transcripts, while cellular RNA was mainly of ribosomal and mitochondrial origin. Preparations were free of contaminating genomic DNA, and coverage correlated remarkably well with viral transcripts locating between promoter and terminator sequence. Notably, besides transcripts of unknown function, most of the 19 viral transcripts identified were associated with immunomodulatory functions, e.g., chemokine-binding or interferon-antagonizing viral proteins. Most abundant in all samples was the *D5L/I1R* gene transcript, encoding an inhibitor of the cellular DNA-dependent protein kinase, which functions as a pattern recognition receptor for DNA in the host cell cytoplasm [15]. This suggests that transcript incorporation may enable host immune suppression even before transcription from the viral genome is initiated and, hence, may represent an adaptive advantage of poxviruses. However, the biological relevance of mRNA incorporated in poxvirus particles during infection remains to be elucidated.

## 2. Materials and Methods

### 2.1. Virus Propagation and Purification

CPXV Brighton Red (BR; ATCC# VR-302) was propagated in HEp-2 (ATCC# CCL-23) and Rat-2 cells (ATCC# CRL-1764) maintained in DMEM supplemented with 2 mM L-Gln and 5% (volume/volume) FCS. CPXV was passaged at least five times in both cell lines prior to virion purification. Purification of intracellular mature virions (IMVs) was done by rate-zonal sucrose gradient centrifugation [16] followed by isopycnic CsCl density gradient centrifugation [17]. For crude purification, the supernatant was centrifuged through a 36% sucrose cushion at 32,900 × *g* and 4 °C for 80 min, and the virus pellet was resuspended in 1 mL of 10 mM Tris pH 9.0. The virus suspension was sonicated for 1 min, layered on a 24–40% continuous sucrose gradient and centrifuged at 26,000 × *g* and 4 °C for 50 min. The virus band was collected and stored at 4 °C, while the pellet was resuspended in 1 mL of 10 mM Tris pH 9.0, sonicated for 1 min and centrifuged through a fresh 24–40% continuous sucrose gradient as described before. The virus bands were pooled and concentrated at 32,900 × *g* and 4 °C for 60 min. The pellet was resuspended in 1 mL of 10 mM Tris pH 9.0 and purified further through a 1.23–1.29 g/mL continuous CsCl gradient at 180,000 × *g* and 4 °C for 4 h. IMV particles with a density of about 1.27 g/mL were aspirated and concentrated through 17 mL of a 36% sucrose cushion as described. The pellet was resuspended in 1 mL of 10 mM Tris pH 9.0 and stored in aliquots at −80 °C. The number of plaque-forming units (PFUs) was determined by a plaque assay as described previously [14]. Control samples containing buffer (10 mM Tris pH 9.0) without virus were carried along during the whole purification procedure.

### 2.2. Sample Preparation for Transcript Analysis

Approximately 2 × 10^9^ PFUs of heat-inactivated (1 h 60 °C) purified CPXV IMV particles or the according volume of heat-inactivated control sample without virus were filled up to 500 µL with 50 mM Tris pH 8.5 and pelleted at 25,000 × *g* and 4 °C for 30 min. The pellet was resuspended in 60 µL of buffer P1 (Qiagen, Hilden, Germany) with or without 100 µg/µL RNase A (Qiagen) and incubated at 37 °C for 30 min. Alternatively, the virus pellet was dissolved in 25 µL of 50 mM Tris pH 8.0 containing 0.25 µg of trypsin (Trypsin Gold MS grade, Promega, Madison, WI, USA) and incubated at RT for 5 min. Trypsin was inactivated by adding Phenylmethylsulfonylfluorid (PMSF; Thermo Fisher Scientific, Rockford, IL, USA) to a final concentration of 1 mM. Virus particles were pelleted at 25,000 × *g* and 4 °C for 10 min and resuspended in buffer P1 for RNase digestion as described. Subsequently, RNA was isolated using the NucleoSpin^®^ RNA II Total RNA Isolation Kit (Macharey-Nagel, Berlin, Germany) according to the manufacturer’s instructions. DNA was eluted in 40 µL of RNase-free water using the high-yield and high-concentration protocol. For digestion of viral DNA, the Ambion Turbo DNA-free™ Kit (Life Technologies, Carlsbad, CA, USA) was used. Briefly, 3 µL of Turbo DNase buffer and 1 µL of Turbo DNase were added to 27 µL of eluate and incubated at 37 °C for 30 min. To stop the digestion, 5 µL of inactivation reagent were added and incubated at RT for 5 min with occasional flipping of the tube. The inactivation reagent was pelleted at 10,000 × *g* for 1.5 min and 10 µL of the supernatant was used for cDNA synthesis. For first-strand synthesis, Super Script II^TM^ reverse transcriptase (Invitrogen, Carlsbad, CA, USA) was used according to the manufacturer’s instructions, including 10 µM random hexamer primers without RNaseOut reagent. For second-strand synthesis, the NEBNext^®^ mRNA Second Strand Synthesis Module (New England Biolabs, Frankfurt/M., Germany) was used according to the manufacturer’s instructions. Double-stranded cDNA was purified using the MinElute Reaction Cleanup Kit (Qiagen) prior to library preparation. During method establishment, removal of RNA by digestion and RNA stability during DNase digestion were verified by spike-in RNA control. Briefly, 10^7^ copies of plasmid-derived Crimean-Congo hemorrhagic fever virus (CCHF) RNA were added before RNase or DNase digestion and assayed for CCHF RNA before and after digestion by real-time PCR [18]. Moreover, the removal of genomic CPXV DNA was analyzed using an OPV-specific real-time PCR (Tables S1–S5) [19].

### 2.3. Next-Generation Sequencing

Double-stranded cDNA was analyzed in the sequencing facility (ZBS 1, Robert Koch Institute, Berlin, Germany) using an Illumina MiSeq machine (Illumina, San Diego, CA, USA). For library preparation, the Nextera^®^ XT DNA Library Preparation Kit (Illumina) was used according to the manufacturers’ instructions. Sequence files are available from the NCBI Sequence Read Archive under the BioProject ID PRJNA401971.

### 2.4. NGS Data Analysis

**Figure 1:**
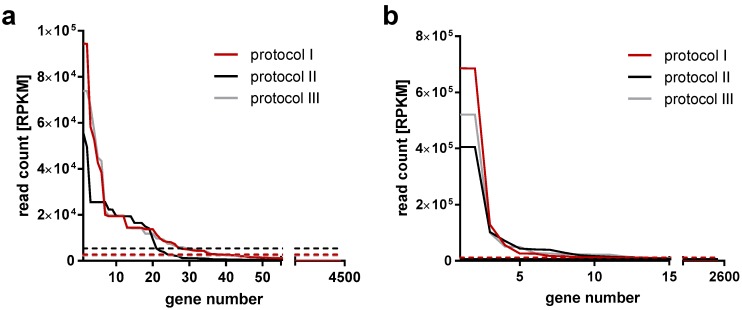
Read distribution in samples analyzed for virion-incorporated transcripts. Highly pure cowpox virus (CPXV) intracellular mature virion (IMV) particles passaged in (**a**) HEp-2 or (**b**) Rat-2 cells were analyzed for transcripts by next-generation sequencing (NGS). Reads mapping to the virus genome and the host genome (human or rat) were sorted by read count (RPKM = reads per kilobase million). Dashed lines: cutoff 90%. Three different protocols were applied for sample preparation: I: RNase digestion, II: no RNase digestion and III: protease and RNase digestion.

Trimmed reads were mapped to both the CPXV BR reference genome and either the *Homo sapiens* reference genome assembly (GRCh38) or the *Rattus norvegicus* reference genome assembly (Rnor6.0), using the split read mapper TopHat2 [20] with standard parameters. Additionally, reads were mapped to reference sequences of human and rat 18S and 28S ribosomal RNA (rRNA), since annotations for these genes are not available in the ENSEMBL database (www.ensembl.org). Reads mapping to the host and the virus genome were identified and extracted using a custom Python script. The number of reads mapping to each gene was counted based on ENSEMBL annotations for the human and rat genomes. This included the mapping to 18S and 28S and annotation from the CPXV BR reference sequence genome, using the tool featureCounts [21]. Read counts were normalized for sequencing depth and gene length (reads per kilobase million, RPKM) and sorted by RPKM. In order to filter out spurious hits, only the top transcripts making up 90% of the RPKM in the sample were considered for further analysis (see Figure 1).

## 3. Results

To analyze the presence of transcripts in CPXV particles without prior sequence knowledge, an NGS-based protocol was established. In summary, RNA outside of virus particles was removed, RNA extracted and the viral DNA digested prior to NGS analysis (Figure S1). To minimize transcripts outside of virions, IMV particles were highly purified by isopycnic CsCl gradient centrifugation. Purity and integrity of particles derived from the purification protocol have been previously confirmed by negative-staining transmission electron microscopy (Figure S2). Moreover, possible residual RNA outside of virus particles was digested with RNase in a first step, which was confirmed by spike-in control during method establishment. Furthermore, it was confirmed by spike-in control that the DNase digestion step after RNA extraction did not result in RNA loss. Transcripts outside of virus particles may also be ribosome-associated, as opposed to free transcripts. Although ribosomes may also be incorporated in virus particles, ribosome-associated transcripts outside of virions may be less accessible to RNase. Hence, a protease digestion was tested prior to RNase digestion. Additionally, the RNase digestion was omitted to estimate the digestion efficiency and to assess transcripts outside of virions. In principle, three protocols were compared: (I) RNase digestion, (II) no RNase digestion and (III) a protease digestion prior to RNase digestion. Control samples without virus particles were carried along during the whole sample preparation, including virion purification, to assess contaminant RNA. The three mentioned protocols were applied in the analysis of transcripts incorporated in CPXV BR passaged in HEp-2 or Rat-2 cells to elucidate possible host-specific transcript incorporation.

### 3.1. Data Overview

Samples showed a maximum coverage of the virus genome of up to 118,673, in contrast to controls with only a maximum coverage of up to 188. This observation was conclusive, since control samples contained buffer without virus particles. Instead, controls displayed a considerable number of host reads. As some virus genes may share homology to host genes, multiple mapping of reads to the host and the virus genome was analyzed. A maximum of nine reads was identified which mapped to both the viral and the according host genome (Table S6). Therefore, multiple mapping reads were considered to be negligible.

Up to about 4500 and 2600 genes were identified in HEp-2- and Rat-2-passaged virions, respectively, including viral and host genes. However, most genes displayed very low read counts. Hence, to define a gene as a transcript hit, the cutoff for each sample was set in a way that hits included 90% of summed normalized read counts (Figure 1). This resulted in 7–27 transcripts in HEp-2-passaged CPXV and 10–22 transcripts in Rat-2-passaged CPXV, depending on the sample preparation protocol. Transcript identifications were sorted by normalized read counts, and the rank was used as a measurement of mRNA amount (Table S7). Notably, exclusively the amount of host transcripts decreased upon RNase digestion. In contrast, the amount of viral transcripts did not decrease upon RNase digestion, indicating that viral transcripts were resistant to digestion and were incorporated in virus particles rather than associated outside of virions. Ribosome-associated transcripts outside of virions should decrease compared to sample preparation without protease treatment or even be no longer identified as transcript. However, with one exception, transcript identifications with protease digestion included all transcript identifications without protease digestion (Table S7).

### 3.2. Incorporation of Viral Transcripts into Virus Particles

**Figure 2:**
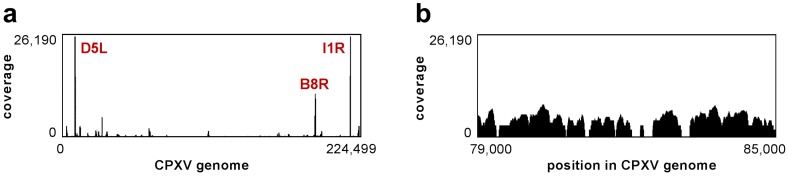
Exemplary coverage across the CPXV genome. Transcripts were analyzed in highly pure CPXV IMV particles using NGS. (**a**) Multiple genes show exceptionally high coverage (red: gene names according to CPXV GRI-90), while some regions displayed no mapping reads at all (**b**). The coverage is shown exemplarily for HEp-2-passaged virions protocol I, but coverage distributions were similar in all samples.

At first sight, the viral genome coverage of each sample displayed conspicuous coverage peaks. The *D5L/I1R* gene (CPXV GRI-90 nomenclature), located in one copy at each end of the genome, displayed by far the highest coverage, followed by the *B8R* gene (CPXV GRI-90 nomenclature). Additionally, viral genome alignments frequently contained sequences without any aligned reads verifying the absence of viral genome contaminations in preparations (Figure 2).

Transcripts identified in at least two sample preparation protocols were considered for further analysis, resulting in 18 and 13 viral transcripts in HEp-2- and Rat-2-passaged virions, respectively (Table 1). Altogether, 19 viral transcripts were identified, including 12 identifications that were shared in CPXV particles passaged in either human or rat cells. Eight viral transcripts were of unknown function, but remarkably, eight transcripts encoded proteins with immunomodulatory function, including two host-range proteins. Furthermore, two viral transcripts belonged to the term transcription and one was associated with viral entry (Table 1).

Sequences with increased coverage mapped remarkably well to viral coding sequences (CDSs). Additionally, the coverage increase was not restricted to annotated CDS, but was also found before and after the respective CDS. This observation made sense in that transcripts were analyzed whose sequence may generally differ from the translated protein sequence as a result of untranslated regions at the 3′ and 5′ ends.

**Figure 3:**
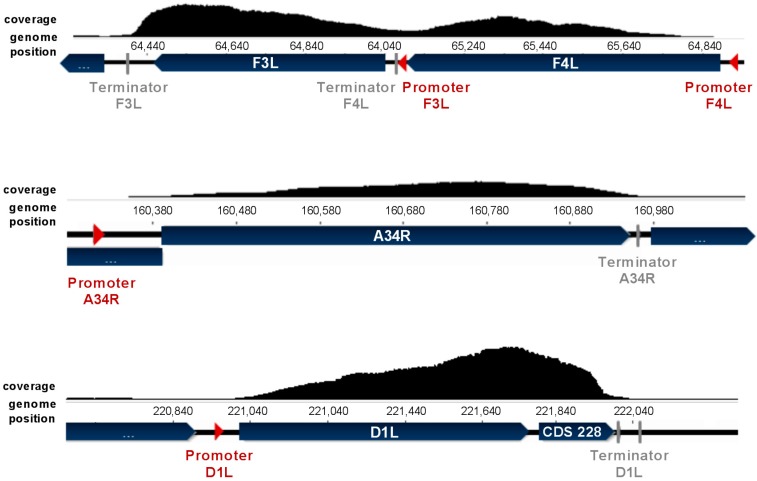
Coverage of viral transcripts between predicted promoter and terminator sequence. The coverage of genes clearly correlates with the predicted promoter [23] and terminator [24] sequences. Pictures show exemplarily the coverage in the HEp-2-passaged CPXV preparation using protocol I.

Transcript reads were expected to localize in between the promoter and the terminator sequence. All identified transcripts, except *B8R*, belonged to the early class of viral genes. Early OPV promoters have been predicted to contain the consensus promoter sequence AAAA---TGAAAA---A [23]. Additionally, viral genes contain single or multiple termination signals which may also be skipped as a result of secondary structures [23]. OPV early genes contain the general functional terminator sequence NTTTTTNT [24]. However, the read distribution was not expected to be completely uniform over transcript length due to method-induced bias; e.g., it is known that non-random binding of random hexamer primers used for cDNA amplification prior to sequencing leads to depleted read counts at the 5′ and 3′ ends [25]. Early viral promoter sequences have been predicted for VACV [23] and were found in nine of the CPXV transcripts identified. These transcripts included the CDS *C8L*, *C10L*, *M1L*, *F3L*, *F4L*, *J5R*, *A34R*, *A36R* and *D1L/I5R*. Terminator sequences were also identified near these transcript 3′ ends. Hence, these genes were used to verify the presence of intact transcripts. The observed coverage fitted remarkably well to the predicted transcript size limited by promoter and terminator sequence. Moreover, coverage distributions showed the expected read depletion at the ends, but mostly also a tendency to 3′ bias, meaning higher coverage towards the mRNA 3′ end (Figure 3). In Figure 3, the coverage of *F3L*, *F4L*, *A34R*, *D1L* and CDS *228* is exemplarily shown. The coverage of adjacent *F3L* and *F4L* showed a clear decrease in between both sequences, indicating separate transcripts. This was underlined by separate promoter and terminator sequences for each transcript. In contrast, *D1L/I5R* and CPXV *228* did not show any coverage decrease in between transcripts but rather a continuously increased coverage. The promoter of the *228* gene is not predicted [23] and a termination sequence in between both transcripts was absent, suggesting a continuous transcription of both CDS.

### 3.3. Incorporation of Host Transcripts

Cytoplasmic 18S and 28S rRNA accounted for the highest amounts of host transcripts in the samples. Additionally, large amounts of mitochondrial (mt) 12S and 16S rRNA were found in virion preparations. Further mt transcripts were identified in Rat-2 samples (*ND1*, *COX1*, *COX3* and *ATP6*). In HEp-2 samples, these mt transcripts were also identified at rather high ranks, but they were not included in the cutoff. The identification of mt transcripts may indicate that the whole mtDNA or even polycistronic mtRNA was purified during sample preparation. Although transfer RNA genes are distributed throughout the mtDNA, only few transfer RNA reads were identified. Hence, it could be excluded that whole mtDNA or even polycistronic mtRNA was purified. Virus particles propagated in HEp-2 cells contained a transcript encoding a predicted microRNA (miRNA 6087) of unknown function (Table S7). However, as this miRNA was also identified as high ranking in the control samples, it was considered a contaminant rather than a virus-incorporated transcript.

## 4. Discussion

The incorporation of transcripts in virus particles may enable the rapid expression of distinct proteins upon infection. The present study is the first one identifying transcripts in poxvirus particles. Since rRNA is the most abundant RNA species in the cell, representing 80–90% of total RNA [26], it was no surprise that large rRNA amounts were detected in all samples. Moreover, rRNA is presumably already contained in virion preparations, as indicated by the identification of ribosomal proteins by proteome analysis of the purified virus stocks [14]. However, rRNA and possibly other contaminating RNA may also be introduced during sample preparation. It should be noted that method-related amplification prior to NGS can result in massive amounts of even the smallest contaminations. This may be underlined by the observation that controls were also dominated by rRNA which did not result from cross-contamination since no rat mt rRNA was detected in rat controls. Cytoplasmic rRNA from humans and rats cannot be distinguished because of sequence identity. In contrast, mt 12S and 16S rRNA display only 79% identity among human and rat, allowing for the distinguishing between contaminant and virion-incorporated rRNA. Moreover, if cross-contamination were an issue, one would expect to identify highly abundant viral transcripts in the control, which was not the case.

RNase digestion was not able to remove rRNA, indicating either an incomplete digestion, virion-incorporated rRNA or rRNA introduced during later sample preparation steps. Incomplete digestion may be ruled out since RNase digestion efficiency was verified by high amounts of RNA spike-in during method establishment. Moreover, the incorporation of rRNA in virus particles may be explained by proximity of ribosomes and mitochondria to virus factories during infection [27]. Taken together, the detected rRNA is presumably a mixture of rRNA contained in virions, associated outside of virions and introduced during sample preparation. However, rat mt rRNA amounts indicate that virion-incorporated rRNA accounts for the largest portion.

Protease digestion prior to RNase digestion did not lead to the expected depletion of transcript identifications. This may be explained by non-ribosome-associated transcripts or incomplete digestion of ribosomes. Additionally, transcripts are possibly digested by RNase although they are ribosome-bound, because only small parts of the transcript are covered by ribosomes. This may be supported by the fact that RNase digestion of ribosome-bound transcripts is the technique underlying ribosome profiling, resulting in small fragments of about 30–31 bp, which are protected by ribosomes during digestion [28].

Apart from rRNA and mt transcripts, exclusively viral transcripts were identified to be incorporated in CPXV particles. These transcripts were not depleted upon RNase digestion, indicating their incorporation in virions. The distinct distribution of reads between promoter and terminator sequences strongly suggests the presence of intact transcripts. Moreover, reads were frequently biased towards the 3′ end of the transcript, which is generally observed in cDNA synthesis with oligo(dT) primers [25]. Controversially, random hexamer primers were used in the present study, which rather tend to induce 5’ bias [29]. However, bias may also be introduced as a result of an mRNA secondary structure, which may lead to the interruption of reverse transcription during cDNA synthesis and early termination [30]. Another explanation for the higher coverages of the transcripts’ 3′ ends may be the presence of transcripts which are partially degraded at the 5′ end, leading to lower 5′ coverage. OPV encode two decapping enzymes which remove the mRNA 5′ cap, resulting in degradation of viral and host mRNA, presumably by the host 5′–3′ mRNA exonuclease *Xrn1* [13]. Decapping promotes translational shutdown of host mRNA and controls dsRNA levels arising from viral transcripts [31]. 

The largest number of transcript with known function was associated with viral immune evasion. Out of eight transcripts encoding immunomodulatory proteins, six were identified in both HEp-2- and Rat-2-passaged virions. These included the DNA-dependent protein kinase inhibitor (DNA-PK) *D5/I1*, the IL-18-binding protein C8, the dsRNA-binding protein F3, the inhibitor of MHC class II antigen presentation A36, the NF-κB inhibitor B13 and the chemokine-binding protein D1/I5. The *I1/D5*-encoding RNA was identified in extraordinarily high amounts. DNA-PK is a large cellular complex which functions as a pattern recognition receptor for cytoplasmic DNA, activating innate immune response. *D5/I1* blocks binding of the DNA-PK to DNA, inhibiting recognition of poxvirus DNA [15].

Viral immunomodulatory genes are generally expressed early during infection in order to rapidly prevent and counteract the host’s antiviral response [22,32]. Moreover, it has been shown that OPVs express an immediate-early class of genes, including 35 genes that are expressed already 0.5–1 h p.i. [33]. Notably, nine transcripts identified in CPXV IMV particles belong to this immediate-early class, including *C14L*, *P2L*, *M1L*, *F3L*, *F4L*, *A34R*, *A36R*, *A47R* and *B13R*. It may be hypothesized that the incorporation of transcripts in virus particles may, at least partially, explain their immediate-early expression. Assarsson and colleagues [33] showed that the immediate-early class of genes is expressed highest during infection, exceeding late gene expression up to 24 h p.i. Therefore, immediate-early transcripts are presumably most abundant during CPXV assembly. Hence, it seems that it can be hypothesized that the incorporation of transcripts is an undirected process occurring proportionally to the intracellular concentration, as was described for CMV [3]. However, the absence of other highly abundant transcripts cannot be explained and there may be also species- and strain-specific differences.

OPV gene expression is organized in a cascade-like manner by genes with early, intermediate and late promoters. Additionally, genes with dual early/late promoters were identified [34]. The vaccinia virus early/late 7.5K gene promoter is particularly well studied. It was shown that the expression of the chloramphenicol acetyltransferase (CAT) under the 7.4K promoter increased late during infection even if the late promoter was deleted, suggesting a reactivation of the early promoter. A similar reactivation of an early viral promoter late in infection was observed for the vaccinia *rpo30* gene [35]. Notably, the CPXV *rpo30* gene (*F4L*) was identified as a virion-incorporated transcript in the present study. Interestingly, it was shown that reactivation of early genes seems to depend on virus assembly and, moreover, on *cis*- and *trans*-acting elements [35,36]. This indicates a rather complex regulation of poxvirus early gene expression. In the context of transcript incorporation, reactivation may serve the production of mRNAs designated for virion incorporation. However, no reactivation of CPXV genes has yet been reported. Poxviruses effectively downregulate host mRNA levels during infection [37], explaining the lack of identified host transcripts other than rRNA and mtRNA in CPXV IMV particles. Nevertheless, if transcript incorporation is proportional to mRNA concentration in the cell, altered mRNA level, e.g., as a result of adaption, may lead to altered transcript incorporation in virions.

Summarized, it can be stated that viral transcripts are incorporated in poxvirus particles, possibly undirected in proportion to their intracellular amount. The biological meaning of these findings, however, remains to be elucidated, e.g., whether poxvirus-incorporated transcripts are released upon infection and result in the expression of intact proteins.

## 5. Conclusions

Transcripts are known to be incorporated in infectious particles of DNA viruses belonging to the families of *Herpesviridae* and *Mimiviridae*, but the presence of transcripts in other DNA viruses, such as poxviruses, has not been thoroughly analyzed yet. By using RNA sequencing of highly purified Cowpox virus virions, this study shows that 19 viral transcripts are incorporated in poxvirus particles, possibly undirected in proportion to their intracellular amount. The function of most of these transcripts is either unknown or related to immunomodulation, including the most abundant transcript originating from the *D5L/I1R* gene that encodes a viral inhibitor of the host cytoplasmic DNA sensing machinery. This suggests that transcript incorporation may enable host immune suppression even before transcription from the viral genome is initiated. However, it remains to be elucidated whether poxvirus-incorporated transcripts are released upon infection and result in the expression of intact proteins.

## Figures and Tables

**Table 1:** Viral transcripts identified in CPXV particles.

Gene *CPXV GRI-90*	Gene *CPXV BR*	Gene *VACV Cop*	Description ^a^	Function	Host ^b^	Expression ^c^
none	*002/228*	no ORF	Uncharacterized protein	unknown	H/R	unknown
*D5L/I1R*	*009/222*	*C16L/B22R*	Inhibitor host DNA-PK ^d^	immune evasion	H/R	unknown
*D6L*	*010*	no ORF	Uncharacterized protein	unknown	H	unknown
*D10L*	*012*	no ORF	Uncharacterized protein	unknown	H/R	unknown
*C6L*	*022*	*C10L*	Uncharacterized protein	unknown	H	early
*C8L*	*024*	no ORF	Interleukin 18-binding	immune evasion	H/R	early
*C10L*	*025*	no ORF	Uncharacterized protein	unknown	H/R	early
*C14L*	*030*	*C6L*	Uncharacterized protein	unknown	H/R	early
*P2L*	*040*	*M2L*	Uncharacterized protein	unknown	H	early
*M1L*	*041*	*K1L*	Interferon antagonist, hr	immune evasion	R	early
*F3L*	*069*	*E3L*	dsRNA-binding protein, hr	immune evasion	H/R	early
*F4L*	*070*	*E4L*	Viral RNA pol 30 kDa	transcription	H	early
*J5R*	*114*	*H5R*	Late transcription factor 4	transcription	H/R	early
*A34*	*168*	*A33R*	EEV glycoprotein	entry	H	early
*A36R*	*171*	*A35R*	Inhibitor MHC class II antigen presentation	immune evasion	H/R	early
*A47R*	*182*	*A44R*	3β-Hydroxysteroid-dehydrogenase/Δ^5-4^-isomerase	immune evasion	H	early
*B8R*	*203*	*B9R*	Uncharacterized protein	unknown	H/R	intermediate
*B13R*	*208*	no ORF	Inhibitor of host NF-κB	immune evasion	H/R	early
*D1L/I5R*	*vCCI*	*B29R/C23L*	Chemokine-binding protein	immune evasion	H/R	early

^a^ according to UniProt, hr = host range; ^b^ cell line used for virus propagation: H = HEp-2, R = Rat-2; ^c^ according to [22]; ^d^ according to [15].

## Supplementary Materials

Supplementary figures and tables are available online at www.mdpi.com/1999-4915/9/10/296/s1.
