# Supplementary material for: A Next-Generation Sequencing Approach Uncovers Viral Transcripts Incorporated in Poxvirus Virions
Source: Viruses. 2017 Oct 13;9(10):296. doi: 10.3390/v9100296 (PMC5691647; doi:10.3390/v9100296)
Supplement: Supplementary file 1 [file viruses-09-00296-s001.pdf]

Supplementary Materials

A Next-Generation Sequencing Approach Uncovers Viral Transcripts Incorporated in Poxvirus Virions

Marica Grossegesse [1], Joerg Doellinger [1,2], Berit Haldemann [3], Lars Schaade [4] and Andreas Nitsche [1*]

Robert Koch Institute, Seestr. 10, 13353 Berlin/Germany

[1] Centre for Biological Threats and Special Pathogens, Highly Pathogenic Viruses (ZBS 1); GrossegesseM@rki.de

[2] Centre for Biological Threats and Special Pathogens, Proteomics and Spectroscopy (ZBS 6); DoellingerJ@rki.de

[3] Methodology and Research Infrastructure, Bioinformatics (MF 1); HaldemannB@rki.de

[4] Centre for Biological Threats and Special Pathogens (ZBS); SchaadeL@rki.de

* Correspondence: NitscheA@rki.de; phone: +49 (0)30 18754 2313

Supplementary Figures:

Figure S1. Sample preparation for the analysis of transcript incorporation in CPXV particles.

Figure S2. Analysis of virion purity and integrity by electron microscopy.

Supplementary Tables:

Table S1. Nucleic acids used for qPCR.

Table S2. Reaction mixture for CCHF qPCR.

Table S3. Thermal profile CCHF qPCR.

Table S4. Reaction mixture for OPV qPCR.

Table S5. Thermal profile OPV qPCR.

Table S6. Overview of reads mapping to virus and host genome.

Table S7. Viral and host transcripts identified in CPXV ranked by normalized read count.

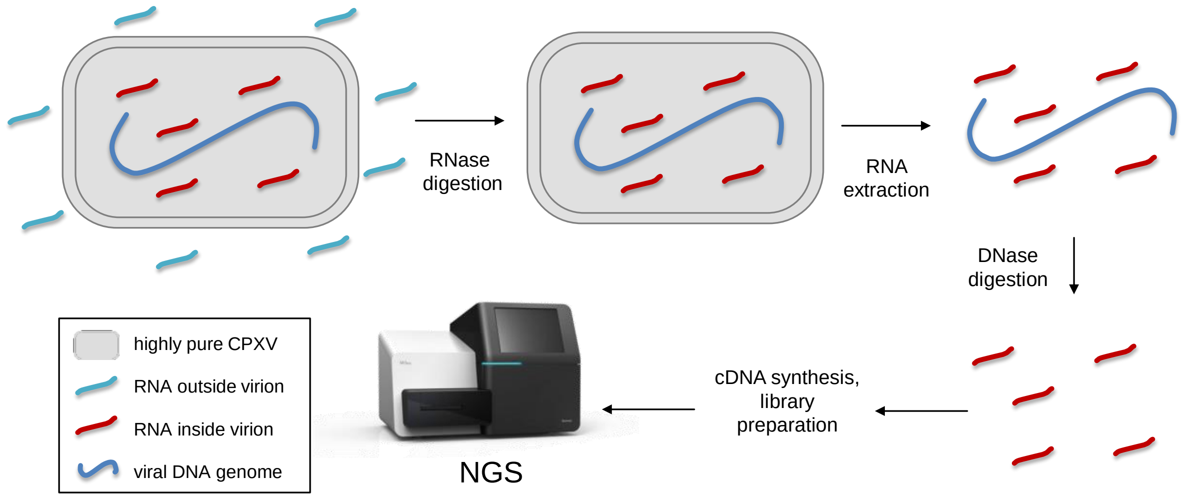

Figure S1. Sample preparation for the analysis of transcript incorporation in CPXV particles. RNA outside of highly pure CPXV particles was digested with RNase prior to RNA extraction. Viral DNA was digested and the leftover RNA analyzed with next-generation sequencing (NGS).

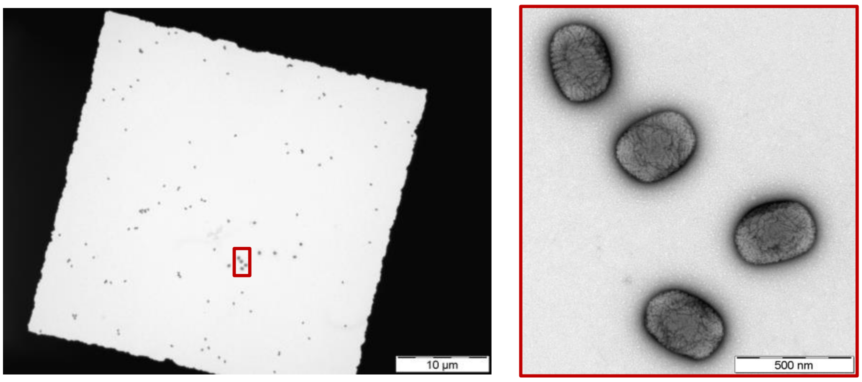

Figure S2. Analysis of virion purity and integrity by electron microscopy. Exemplary pictures of CsCl purified CPXV BR stock (HEp-2-adapted). No protein clouds are detectable outside of virions and particles appear fully intact.

Table S1. Nucleic acids used for qPCR.

Name	Sequence (5′> 3′)	Application
P4A F	TAA TAC TTC gAT TgC TCA TCC Agg	Poxvirus reference sequence [1]
P4A R	ACT TCT CAC AAA Tgg ATT TgA AAA TC	
P4A probe	Fam-TCC TTT ACg TgA TAA ATC AT-MGB	
CCforSE01	CAA ggg gTA CCA AgA AAA TgA AgA AGG C	Detection of CCHF RNA [2]
CCrevSE02	gCC ACA ggg ATT gTT CCA AAg CAg AC	
CCprobeSE01	FAM-TgT CAA CAC AgC Agg gTg CAT gTA gAT-BHQ1	
CCprobeSE03	FAM-TgT AAg CAC ggC Agg gTg CAT gTA AAT-BHQ1	
CCprobeSE04	FAM-ATC TAT ATg CAC CCC gCT gTg TTA ACA-BHQ1	
CCprobeSE0A	FAM-ACT CCA ATg AAg Tgg ggg AAg AAg CT-BHQ1	

Table S2. Reaction mixture for CCHF qPCR.

Reagent	Volume per reaction [µl]
H_2O (PCR-grade)	3.5
RT buffer	2.5
Enhancer	1
CCforSE01	1.50
CCrevSE02	1.50
CCprobeSE01	0.25
CCprobeSE03	0.25
CCprobeSE04	0.25
CCprobeSE0A	0.25
Enzyme Mix	1.00

Table S3. Thermal profile CCHF qPCR.

Temperature [°C]	Time [s]	Cycle
45	900	1 x
95	600	1 x
94	20	45 x
59	30	

Table S4. Reaction mixture for OPV qPCR.

Reagent	Volume per reaction [µl]
H_2O (PCR-grade)	14.15
10 x PCR buffer	2.5
$MgCl_2$ (50 mM)	2
dNTPs (2.5mM)	2
F primer (10 µM)	0.75
R primer (10 µM)	0.75
Probe (10 µM)	0.25
Platinum Taq (5 U/µl)	0.1

Table S5. Thermal profile OPV qPCR.

Temperature [°C]	Time [s]	Cycle
95	600	1 x
95	10	45 x
60	35	

1. Schroeder, K.; Nitsche, A. Multicolour, multiplex real-time pcr assay for the detection of human-pathogenic poxviruses. *Mol Cell Probes* **2010**, *24*, 110-113.
2. Wolfel, R.; Paweska, J.T.; Petersen, N.; Grobbelaar, A.A.; Leman, P.A.; Hewson, R.; Georges-Courbot, M.C.; Papa, A.; Gunther, S.; Drosten, C. Virus detection and monitoring of viral load in crimean-congo hemorrhagic fever virus patients. *Emerg Infect Dis* **2007**, *13*, 1097-1100.

Table S6. Overview of reads mapping to virus and host genome.

Sample	Protocol	Viral reads	Host reads	Mean genome coverage[1]	Max. coverage[2]	Multiple mapping[3]
CPXV BR, HEp-2 adapted	I	3.3E+05	6.4E+05	201.4	25,172	4
	II	1.0E+05	1.1E+06	49.9	7,134	4
	III	2.6E+05	5.7E+05	141.6	17,081	1
CPXV BR, Rat-2 adapted	I	8.4E+05	4.7E+04	489.3	118,673	0
	II	6.7E+05	3.6E+05	578.6	114,124	1
	III	6.3E+05	1.4E+05	504.7	106,390	0
Control HEp-2 adapted	I	7.1E+03	4.1E+05	3.6	188	2
	II	1.5E+03	2.6E+05	0.6	51	0
	III	6.5E+03	2.8E+05	2.7	121	9
Control Rat-2 adapted	I	5.5E+02	9.2E+03	0.4	51	0
	II	8.3E+02	3.4E+04	0.6	118	1
	III	5.4E+02	2.4E+04	0.4	79	0

I: RNase digestion, II: no RNase digestion, III: protease and RNase digestion

[1]viral genome coverage [2]max. coverage viral genome [3]reads mapping to virus and host genome

Table S7. Viral and host transcripts identified in CPXV ranked by normalized read count.

Rank	HEp_I	HEp_II	HEp_III	Rat_I	Rat_II	Rat_III
1	D5L/I1R[1]	18S rRNA	D5L/I1R	D5L/I1R	D5L/I1R	D5L/I1R
2	18S rRNA	28S rRNA	18S rRNA	B8R	mt 12S rRNA	B8R
3	28S rRNA	mt 12S rRNA	28S rRNA	B13R	18S rRNA	18S rRNA
4	B8R	mt 16S rRNA	mt 16S rRNA	C14L	B8R	B13R
5	mt 16S rRNA	D5L/I1R	B8R	C10L	mt 16S rRNA	mt 12S rRNA
6	C10L	C10L	mt 12S rRNA	J5R	B13R	C10L
7	mt 12S rRNA	B8R	C10L	D1L/I5R	002/228[1]	C14L
8	D10L		D10L	18S rRNA	C10L	mt 16S rRNA
9	F3L		002/228[1]	M1L	J5R	J5R
10	D1L/I5R		F3L	A36R	C14L	D1L/I5R
11	002/228[1]		C8L		D1L/I5R	002/228[1]
12	C8L		D1L/I5R		ND1	D10L
13	B13R		C14L		28S rRNA	M1L
14	C14L		B13R		COX3	ND1
15	J5R		C6L		D10L	A36R
16	F4L		F4L		ATP6	C8L
17	C6L		D6L		COX1	COX3
18	D6L		MIR6087		B2R	F3L
19	A36R		J5R		M1L	P2L
20	A34R		A36R		F3L	
21	MIR6087		A34R		C8L	
22	P2L		P2L		A36R	
23	A47R		C13L			
24	B12R		M1L			
25			B10R			
26			A47R			
27			D8L			

I: RNase digestion, II: no RNase digestion, III: protease and RNase digestion
Redundant genes were summarized. white: viral; grey: host; red: ribosomal RNA (mt = mitochondrial)
Gene names acc. CPXV BR
